# Large-cell neuroendocrine carcinoma of the bile duct: Case report of surgical treatment and adjuvant chemotherapy of 2 cases

**DOI:** 10.1097/MD.0000000000039848

**Published:** 2024-09-27

**Authors:** Chang Ho Seo, Ho Joong Choi

**Affiliations:** a Department of Surgery, Bucheon St. Mary’s Hospital, College of Medicine, Catholic University of Korea, Seoul, Republic of Korea; b Department of Surgery, Division of Hepatobiliary-Pancreas Surgery and Liver Transplantation, Seoul St. Mary’s Hospital, College of Medicine, Catholic University of Korea, Seoul, Republic of Korea.

**Keywords:** adjuvant chemotherapy, case report, large-cell neuroendocrine carcinoma, neuroendocrine carcinoma

## Abstract

**Rationale:**

Neuroendocrine carcinoma originating from extrahepatic bile duct is very rare, and only a few cases have been reported. Because of its scarcity of incidence, not much is known about the disease but for its aggressiveness and poor prognosis.

**Patient concerns:**

In this report, we present 2 cases of large cell neuroendocrine carcinoma (LCNEC) originating from extrahepatic bile duct. Case 1: a 60-year-old woman presented with jaundice but no abdominal pain. Case 2: a 67-year-old man also presented with jaundice, along with abdominal discomfort and appetite loss.

**Diagnoses:**

Case 1: LCNEC with a focal adenocarcinoma component (pT2aN1M0, pStage IIIB). Case 2: LCNEC with a focal adenocarcinoma component (pT1N1M0, pStage IIB).

**Interventions:**

Case 1: the patient underwent left hepatectomy and caudatectomy with hepaticojejunostomy, followed by 6 cycles of adjuvant chemotherapy (etoposide and cisplatin). Case 2: the patient underwent laparoscopic pylorus–preserving pancreatoduodenectomy, followed by 6 cycles of adjuvant chemotherapy (etoposide and cisplatin).

**Outcomes:**

Case 1: liver metastasis was detected 6 months postoperatively, and despite multiple chemotherapy regimens, the patient died 24 months post-surgery. Case 2: liver metastasis was detected 23 months postoperatively. The patient is still alive 36 months post-surgery after receiving multiple chemotherapy regimens and radiotherapy.

**Lessons:**

Given the rarity of LCNEC, it is essential to continue collecting and reporting additional case studies to build a more comprehensive understanding of the disease. Although the prognosis for LCNEC is generally poor, the use of a multidisciplinary approach and further research will be critical in developing more effective treatment strategies in the future.

## 1. Introduction

Large-cell neuroendocrine carcinoma (LCNEC) of the extrahepatic bile duct is an exceedingly rare and aggressive neoplasm.^[1,2]^ Due to its low incidence, there is a scarcity of case reports and a limited understanding of its clinical behavior, optimal treatment strategies, and prognosis.^[3,4]^ Most of the available literature highlights the aggressive nature and poor prognosis associated with LCNEC, emphasizing the need for early detection and comprehensive treatment.^[5,6]^ Neuroendocrine tumors of the biliary tract are uncommon, with the majority of reported cases originating from the gallbladder or the intrahepatic bile ducts.^[2,7]^ LCNEC, a high-grade variant of neuroendocrine carcinoma, is characterized by large cells with abundant cytoplasm, prominent nucleoli, and high mitotic activity. The rarity of LCNEC in the extrahepatic bile duct poses significant diagnostic and therapeutic challenges for clinicians.

Herein, we present 2 cases of LCNEC originating from the extrahepatic bile duct. Both patients underwent successful surgical resection followed by adjuvant chemotherapy. LCNEC is an exceptionally rare type of cancer, particularly within the bile duct, with limited established guidelines for adjuvant chemotherapy due to its rarity. Through this report, we have analyzed 2 cases, providing detailed clinical results and long-term follow-up data. We aim to contribute to the existing literature by providing detailed clinical and pathological insights, discussing therapeutic approaches, and emphasizing the importance of a multidisciplinary approach for this rare malignancy.

## 2. Case presentation

### 2.1. Case 1

**Figure 1.**
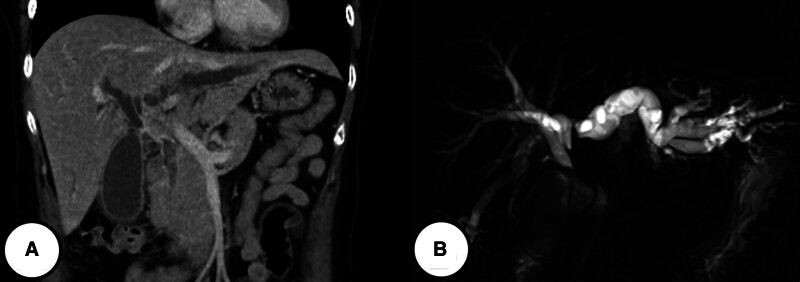
Abdominal image scan of the first case patient showing approximately 3.6 cm irregular enhancing wall thickening in hepatic hilum, probable periductal infiltrating type Klatskin tumor (Bismuth IIIa). (A) Abdominal computed tomography scan. (B) Magnetic resonance cholangiopancreatography scan.

**Figure 2.**
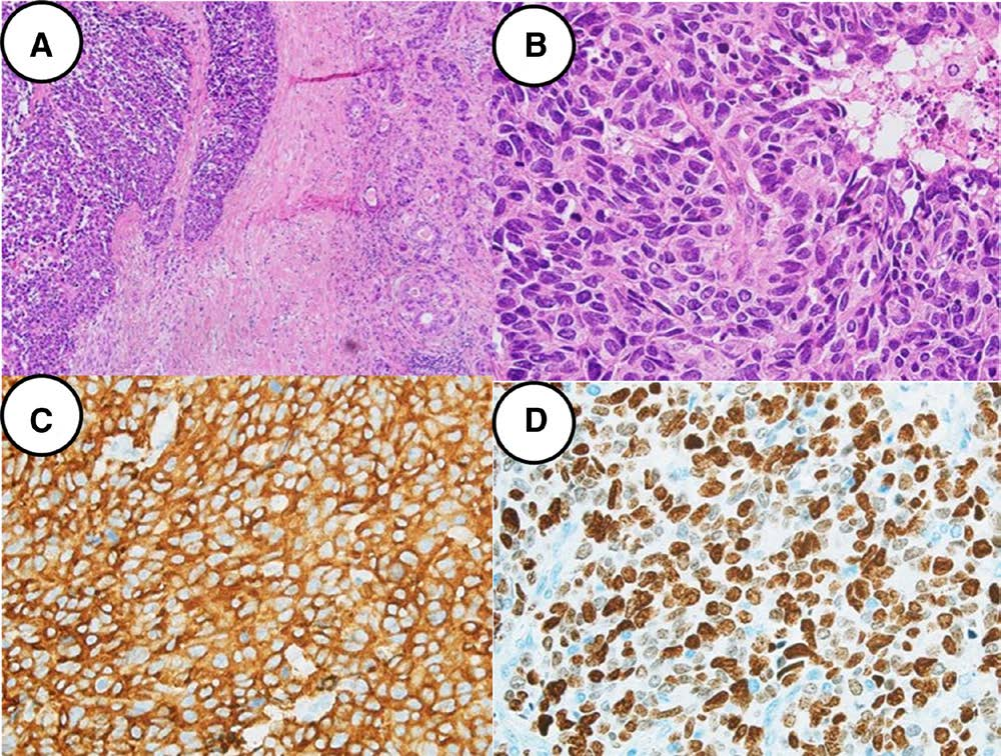
Microscopic histopathologic report of the first case patient. (A) The tumor consists of compactly packed neuroendocrine carcinoma cells (left side) and a small proportion of gland-forming adenocarcinoma cells (right side, about 10% of the entire tumor area; H&E, ×100). (B) Neuroendocrine carcinoma cells are large sized. They have considerable amount of cytoplasm and vesicular nuclei. Mitoses are frequently seen (arrows; H&E, ×400). The tumor shows necrosis (right upper side). (C) On immunohistochemical staining, the tumor cells are positive for synaptophysin (×400). (D) Ki-67 proliferation index is about 90% (×400). H&E = hematoxylin and eosin.

A 60-year-old female patient presented to the emergency room of Seoul St. Mary’s Hospital with jaundice. She did not report abdominal pain, nausea, or vomiting but complained of pruritus. The patient’s medical history revealed no prescribed medications, although she had recently taken herbal supplements and health products. She denied any significant medical history, previous surgeries, or family history of liver disease or cancer. Upon admission, the blood test results were as follows: white blood cells, 7810/mm³; hemoglobin, 12.7 g/dL; platelets, 351,000/mm³; aspartate aminotransferase, 189 U/L; alanine aminotransferase, 103 U/L; alkaline phosphatase, 992 U/L; total bilirubin, 18.7 mg/dL; direct bilirubin, 17.1 mg/dL; carcinoembryonic antigen, 1.26 ng/mL; carbohydrate antigen 19-9, 65.99 U/mL. Imaging studies, including computed tomography (CT) and magnetic resonance imaging, revealed approximately 3.6 cm irregular enhancing wall thickening in the hepatic hilum, suggesting a probable periductal infiltrating type Klatskin tumor (Bismuth IIIa) (Fig. 1). A PET-CT scan showed no metastasis to other organs except the biliary tract. The endoscopic retrograde cholangiopancreatography procedure was performed, revealing strictures from the common hepatic duct to the hilum with the discharge of pus during the procedure. Biopsies were taken from the stricture site, and 1 endoscopic retrograde biliary drainage was inserted into both the left and right bile ducts. The biopsy results indicated malignancy consistent with small-cell carcinoma, and the patient was referred for surgical resection. The patient underwent left hepatectomy and caudatectomy with hepaticojejunostomy. The final pathology report revealed neuroendocrine carcinoma, large cell type, with an adenocarcinoma component (about 10%). The tumor was staged as pT2aN1M0, pStage IIIB, measuring 4.7 × 2.5 × 2.2 cm, with an infiltrating gross type. Lymph node metastasis was present in 1 out of 15 nodes (pN1), and the resection margins of both the proximal bile duct and distal bile duct were very close (<0.1 cm). The mitotic count was 29/10 high power fields. Immunohistochemistry was positive for chromogranin, synaptophysin, and cluster of differentiation (CD56), with a Ki-67 index of 80% (Fig. 2). Postoperatively, the patient recovered without complications and was discharged in good condition on the 9th day. Adjuvant chemotherapy with etoposide and cisplatin for 6 cycles was administered. Six months after surgery, multiple tiny hepatic metastases were detected. The patient received 8 cycles of 5-fluorouracil (5-FU) and oxaliplatin, 4 cycles of 5-FU and irinotecan, and 2 cycles of 5-FU and cisplatin. Despite these treatments, the disease progressed, and the patient expired 24 months after surgery.

### 2.2. Case 2

**Figure 3.**
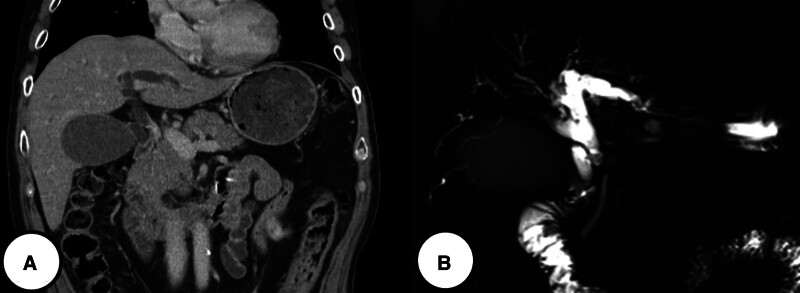
Abdominal image scan of the second case patient showing biliary obstruction just below cystic duct confluence including 2 cm length mass. (A) Abdominal computed tomography scan. (B) Magnetic resonance cholangiopancreatography scan.

**Figure 4.**
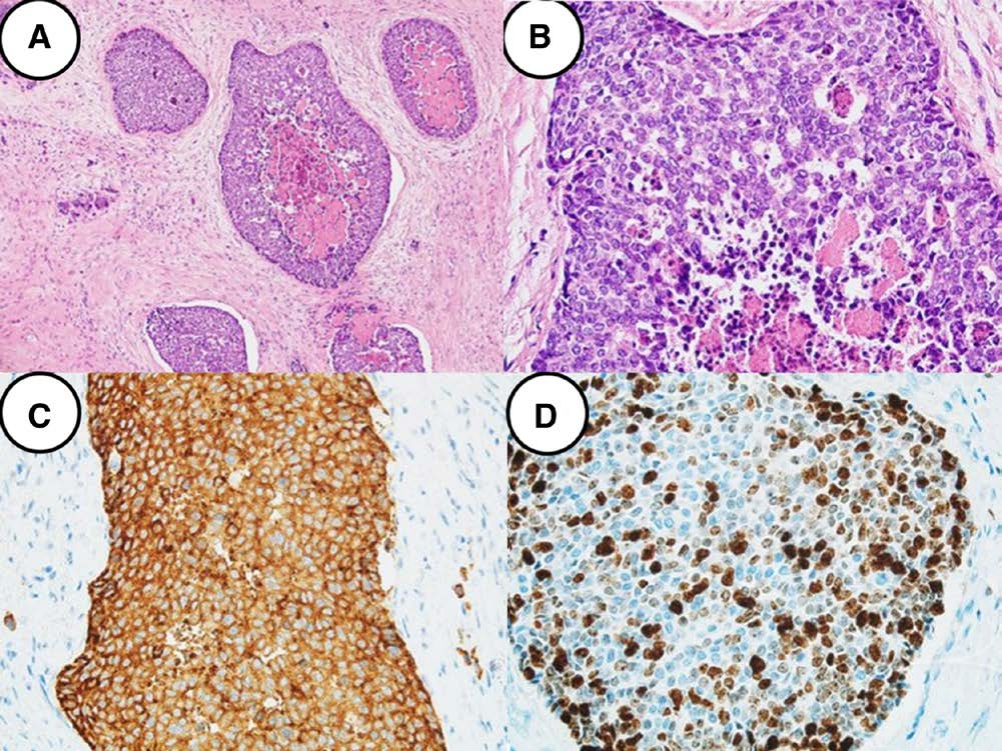
Microscopic histopathologic report of the second case patient. (A) The tumor cells are arranged in large nests containing central necrosis in background of fibrotic stroma (H&E, ×100). (B) The tumor cells are large sized and have angulated nuclei with salt and pepper patterned chromatin (H&E, ×400). (C) On immunohistochemical staining, the tumor cells are positive for synaptophysin (×400). (D) Ki-67 proliferation index is about 60% (×400). H&E = hematoxylin and eosin.

A 67-year-old male patient was admitted to Seoul St. Mary’s Hospital with upper abdominal discomfort and jaundice, which had been occurring for 3 weeks. He reported a feeling of fullness in the abdomen, which affected his appetite, but denied any abdominal tenderness. The patient had no significant medical history, medications, or previous surgeries, and he denied any family history of cancer. Initial blood tests showed the following: white blood cells, 9530/mm³; hemoglobin, 14.2 g/dL; platelets, 292,000/mm³; aspartate aminotransferase, 369 U/L; alanine aminotransferase, 650 U/L; alkaline phosphatase, 574 U/L; total bilirubin, 16.5 mg/dL; direct bilirubin, 14.8 mg/dL; carcinoembryonic antigen, 1.39 ng/mL; and carbohydrate antigen 19-9, 75.7 U/mL. Imaging studies, including CT and magnetic resonance imaging, revealed a distal common bile duct cancer, approximately 2 cm in length, just below the cystic duct confluence, causing biliary obstruction (Fig. 3). Endoscopic retrograde cholangiopancreatography procedure was performed, revealing a distal common bile duct stricture. An endobiliary biopsy was taken, and an endoscopic retrograde biliary drainage was inserted. A PET-CT scan showed no metastasis to other organs except the biliary tract. The biopsy results indicated biliary intraepithelial neoplasia, high grade, and the patient was referred for surgery. The patient underwent a laparoscopic pylorus-preserving pancreaticoduodenectomy. Postoperatively, bile leakage was observed at the hepaticojejunostomy site, and a percutaneous transhepatic biliary drainage was inserted, which was removed 2 months later. The final pathology report revealed LCNEC with a focal adenocarcinoma component (5%), staged as pT1N1M0, pStage IIB. The tumor measured 1.9 × 1.4 × 0.4 cm, with an infiltrative gross type, grayish-white color, and firm consistency. Mitosis was observed at 2/10 high power fields. Lymph node metastasis was present in 1 out of 16 nodes (pN1), and there was no involvement of the resection margins. Immunohistochemistry showed positivity for chromogranin, synaptophysin, CD56, and cytokeratin 7 and focal positivity for caudal-type homeobox transcription factor 2, with a Ki-67 index of 60% in hot spot areas (Fig. 4). Postoperatively, the patient received adjuvant chemotherapy with etoposide and cisplatin for 6 cycles. At 23 months post-operation, liver recurrence was detected. The patient underwent radiotherapy and received additional chemotherapy, including etoposide + platinum (2 cycles), 5-FU + oxaliplatin (9 cycles), and 5-FU + irinotecan (3 cycles). After 36 months post-operation, the patient remains alive but exhibits hepatic and bone metastases, indicative of disease progression.

## 3. Discussion

Neuroendocrine carcinoma of the bile duct, particularly LCNEC, represents an exceptionally rare entity within oncological pathology.^[1–3]^ Due to its infrequency, comprehensive understanding and standardized treatment protocols remain elusive.^[4,5]^ In this context, the presentation of 2 consecutive cases of LCNEC from a single institution underscores the exceptional nature of these occurrences.

LCNEC is characterized by large tumor cells with abundant cytoplasm, prominent nucleoli, and a high mitotic rate. These tumors exhibit aggressive behavior and often result in a poor prognosis. Immunohistochemical staining is pivotal for diagnosis, commonly revealing positivity for neuroendocrine markers such as chromogranin, synaptophysin, and CD56, along with a high Ki-67 index indicating a high proliferation rate.^[8]^

The optimal treatment strategy for LCNEC remains undefined due to the limited number of reported cases.^[7]^ Surgical resection remains the primary treatment modality for localized disease, aiming for complete tumor removal with negative margins. However, the role of adjuvant therapy is less clear, though it is often employed in an attempt to control microscopic residual disease and prevent recurrence.^[9]^ The second patient’s relatively longer survival with disease control, despite the initial disease presentation, underscores the importance of tailored treatment regimens based on individual patient response. According to our investigation, there are only 7 case reports on the treatment of LCNEC. We have summarized these findings, along with those from 7 previously reported cases, in Table 1. Previous reports include follow-up periods of just over a year at most. In contrast, our report provides detailed follow-up and treatment outcomes of relatively long-term treatment spanning 24 to 36 months, offering a comprehensive comparison with previously reported cases. Given the absence of well-established chemotherapy treatment protocols for LCNEC, determining adjuvant treatment plans through a multidisciplinary approach is currently considered optimal.

**Table 1:** Summary of reported cases of LCNECs of the common bile ducts.

Reference	Age (yr)	Sex	Pathology	Location	Size (cm)	Treatment	Survival
Sato et al^[1]^	68	Male	LCNEC + AD	Bd	2	Resection and CTx	3 mo, dead
Demoreuil et al^[2]^	73	Male	LCNEC + AD	Bh	3	Resection and CTx	12 mo, dead
Sasatomi et al^[3]^	76	Male	LCNEC	Bh	5	Resection	1 mo, dead
Ninomiya et al^[4]^	75	Female	LCNEC	Bm	3	Resection	14 mo, alive
Park et al^[5]^	75	Female	LCNEC	Bm	2.7	Resection and CTx	12 mo, dead
Murakami et al^[6]^	79	Male	LCNEC + AD	Bh	2.9	Resection	3 mo, dead
Park and Jeon^[7]^	59	Male	LCNEC	Bh-Bm	4.5	Resection + CCRT	12 mo, alive
Current-1	60	Female	LCNEC + AD	Bh	4.7	Resection and CTx	24 mo, dead
Current-2	67	Male	LCNEC + AD	Bd	1.9	Resection and CTx + RT	36 mo, alive

AD = adenocarcinoma, Bd = distal portion of the bile duct, Bh = hilar portion of the bile duct, Bm = mid portion of the bile duct, CCRT = concurrent chemoradiotherapy, CTx = chemotherapy, LCNEC = large-cell neuroendocrine carcinoma.

In our reported cases, both patients underwent successful surgical resection followed by adjuvant chemotherapy with etoposide and cisplatin. This regimen is frequently used in the treatment of neuroendocrine carcinomas, given their sensitivity to platinum-based chemotherapeutic agents,^[9]^ as seen in previous cases summarized in Table 1, where similar regimens were predominantly utilized. The presence of an adenocarcinoma component, as observed in both of our cases, further complicates the pathological landscape, suggesting a mixed differentiation that may influence treatment responses and outcomes. The first patient, however, experienced disease progression with hepatic metastases detected 6 months post-operation, highlighting the aggressive nature of LCNEC despite the initial treatment. In contrast, the second patient, although initially showing evidence of disease, has demonstrated a longer survival with disease control, emphasizing the variability in disease progression and response to treatment.

Immunohistochemically, the consistent expression of neuroendocrine markers such as chromogranin, synaptophysin, and CD56 in both cases underscores the neuroendocrine origin of these tumors. The high Ki-67 proliferation index reflects the aggressive nature of LCNEC, correlating with the rapid disease progression observed in both patients.

In summary, LCNEC of the bile duct is a rare and aggressive malignancy with a poor prognosis. Our report of 2 consecutive cases underscores the need for further research to establish standardized treatment protocols. Continued documentation and analysis of such rare cases are essential to enhance understanding and improve management strategies for this challenging disease.

## 4. Final diagnosis

LCNEC with an adenocarcinoma component.

## 5. Conclusion

Neuroendocrine carcinoma of the extrahepatic biliary tracts is a very rare and highly malignant disease with a poor prognosis. To establish a definite treatment approach, more cases should be found and reviewed. Until then, a multidisciplinary approach could improve the prognosis for this neoplasm.

## Author contributions

**Data curation:** Chang Ho Seo, Ho Joong Choi.

**Investigation:** Chang Ho Seo, Ho Joong Choi.

**Methodology:** Chang Ho Seo, Ho Joong Choi.

**Visualization:** Chang Ho Seo, Ho Joong Choi.

**Writing – original draft:** Chang Ho Seo.

**Writing – review & editing:** Chang Ho Seo, Ho Joong Choi.

**Conceptualization:** Ho Joong Choi.

**Project administration:** Ho Joong Choi.

**Supervision:** Ho Joong Choi.
